# French recommendations for the management of Behçet’s disease

**DOI:** 10.1186/s13023-020-01620-4

**Published:** 2021-02-24

**Authors:** Isabelle Kone-Paut, Stéphane Barete, Bahram Bodaghi, Kumaran Deiva, Anne-Claire Desbois, Caroline Galeotti, Julien Gaudric, Gilles Kaplanski, Alfred Mahr, Nicolas Noel, Maryam Piram, Tu-Anh Tran, Bertrand Wechsler, David Saadoun, Collaborators, Achille Aouba, Marc Bayen, Patrice Cacoub, Raphael Darbon, Alban Deroux, Brigitte Granel, Mohamed Hamidou, Laurent Kodjikian, Marc Lambert, Dan Lipsker, Céline Marsaud, François Maurier, Isabelle Melki, Tristan Mirault, Antoine Parrot, Aurelie Plessier, Grégory Pugnet, Karine Retornaz, Sophie Riviere, Karim Sacre, Philippe Seksik, Damien Sene, Pascal Seve, Stephanie Tellier, Benjamin Terrier

**Affiliations:** 1 Pediatric Rheumatology and CEREMAIA, Bicêtre Hospital APHP, University of Paris Sud Saclay, Le Kremlin-Bicêtre, France; 2 Unit of Dermatology, DMU3ID, Groupe Hospitalier Pitié-Salpêtrière, AP-HP, and Inflammation-Immunopathology-Biotherapy Department (DHU i2B), INSERM-UMRS 959, Sorbonne Universités, Paris, France; 3 Department of Ophthalmology, CRMR OPHTARA, IHU FOReSIGHT, Pitié-Salpêtrière Hospital, APHP, Sorbonne University, Paris, France; 4 Department of Pediatric Neurology, National Referral Center for Rare Inflammatory Brain and Spinal Diseases, Assistance Publique-Hopitaux de Paris, University Hospitals of Paris-Saclay, Bicêtre Hospital, Paris, France; 5 Inserm UMR1184, Immunology of Viral Infections and Autoimmune Diseases, University Paris Saclay, Le Kremlin-Bicêtre, France; 6 UPMC Université Paris 06, Inserm UMR S 959, Immunology Immunopathology Immunotherapy (I3), Sorbonne Universités, 75005 Paris, France; 7 Biotherapy (CIC-BTi) and Inflammation-Immunopathology-Biotherapy Department (DHU i2B), Hôpital Pitié-Salpêtrière, AP-HP, 75651 Paris, France; 8 AP-HP groupe hospitalier Pitié-Salpêtrière, Department of Internal Medicine and Clinical Immunology, centre national de référence maladies autoinflammatoires et amylose inflammatoire, centre national de références maladies autoimmunes systémiques rares, 75013 Paris, France; 9 Department of Vascular Surgery, Pitié-Salpétrière Hospital, Assistance Publique Hôpitaux de Paris, Sorbonne Université, Paris, France; 10 Internal Medicine and Clinical Immunology Department, Hôpital de la Conception, Aix-Marseille Université, Marseille, France; 11 Clinic for Rheumatology, Kantonsspital St Gallen, St Gallen, Switzerland; 12 Assistance Publique-Hôpitaux de Paris, Service de Médecine Interne et Immunologie Clinique, CHU Bicêtre, Le Kremlin Bicêtre, France; 13 INSERM, UMR 1184, Immunologie des Maladies Virales et Autoimmunes, Université Paris Saclay, Le Kremlin Bicêtre, France; 14 CEA, DSV/iMETI, Division of Immuno-Virology, IDMIT, Fontenay aux Roses, France; 15 Pediatric Dermatology, CHU Sainte Justine Research Centre, CHU Sainte Justine, University of Montreal, Montreal, Canada; 16 Department of Pediatrics, Nîmes University Hospital, INSERM U1183, Montpellier-Nîmes University, Nîmes, France

**Keywords:** Behçet’s disease, Recommendation, Management

## Abstract

Behçet’s disease (BD) is a systemic variable vessel vasculitis that involves the skin, mucosa, joints, eyes, arteries, veins, nervous system and gastrointestinal system, presenting with remissions and exacerbations. It is a multifactorial disease, and several triggering factors including oral cavity infections and viruses may induce inflammatory attacks in genetically susceptible individuals. BD vasculitis involves different vessel types and sizes of the vascular tree with mixed-cellular perivascular infiltrates and is often complicated by recurrent thrombosis, particularly in the venous compartment. Several new therapeutic modalities with different mechanisms of action have been studied in patients with BD. A substantial amount of new data have been published on the management of BD, especially with biologics, over the last years. These important therapeutic advances in BD have led us to propose French recommendations for the management of Behçet’s disease [Protocole National de Diagnostic et de Soins de la maladie de Behçet (PNDS)]. These recommendations are divided into two parts: (1) the diagnostic process and initial assessment; (2) the therapeutic management. Thirty key points summarize the essence of the recommendations. We highlighted the main differential diagnosis of BD according to the type of clinical involvement; the role of genetics is also discussed, and we indicate the clinical presentations that must lead to the search for a genetic cause.

## Introduction

### Summary

#### Summary for the treating physician

Behçet’s disease is a vascularity of vessels of all calibers that affects the arterial and venous areas. It preferentially affects subjects of young age, most often from 10 to 45 years, and affects both men and women. A first outbreak after 50 years of age is rare. Behçet’s disease is ubiquitous but most frequent in patients originating in the Mediterranean basin, the Middle East, and Asia. The causes of the disease are unknown.

Because of the absence of specific biological criteria, the diagnosis is essentially clinical. The diagnostic criteria make it possible to establish the diagnosis with good sensitivity and specificity. Behçet’s disease develops by outbreaks. The primary manifestations are:

- Mucocutaneous, associating a recurrent and sometimes genital buccal aphthosis (aphthosis termed bipolar), pseudo-folliculitis (or folliculitis), and cutaneous hyperreactivity (pathergy test), and more rarely an erythema nodosum.
- Articular, with inflammatory, recurrent, and asymmetrical arthralgia and/or oligoarthritis affecting the large joints.
- Ocular, being manifested by inflammatory ocular outbreaks (uveitis) involving all segments of the eye (panuveitis). Posterior involvement is quasi-constant in case of ocular damage and exposes the patient to the risk of blindness.
- Vascular:
- Superficial venous thromboses are transitory and migratory. The deep vein thromboses may affect all the venous areas.
- Arterial damage manifests by thromboses or aneurysms, is often multiple, and localizes in the pulmonary vessels, the aorta, or the peripheral arteries.
- Neurological involvement varies, sometimes preceded by fever and headaches, and meningitis and meningoencephalitis are at the forefront.
- Digestive involvement is similar to that of chronic inflammatory bowel diseases.

The prognosis of Behçet’s disease is variable from one patient to another but the disease may be serious. Mucocutaneous damage may be very debilitating and profoundly affect the patient’s quality of life. Ocular damage engages the visual acuity with a non-negligible risk of blindness. Neurological damage entails exposure to the risk of serious neurological sequelae. Finally, vascular damage, particularly arterial, is serious and remains the principal cause of death in patients affected by Behçet’s disease.

Specialized multi-disciplinary care in an expert center is necessary for this rare disease with very polymorphic expression and requiring extended treatments and lifelong follow-up. The drug treatments for Behçet’s disease essentially depend on the clinical manifestations and target the abatement of inflammation.

Colchicine is the first-choice treatment of mucocutaneous and articular involvement. The most serious, ocular, vascular, or neurological involvements require immunomodulating treatment, most often associating systemic corticosteroid therapy with immunosuppressants and biotherapy (anti-TNFα), depending on the indication.

The use of anticoagulants in treating vascular involvement is disputed but remains recommended; inflammation of the vascular walls is the main cause of Behçet’s disease-related thrombosis. The prescription of aspirin at an antiaggregating dose is considered upon stenosizing the arterial damage.

In its serious and/or complicated forms, Behçet’s disease is categorized as a life-long disorder, with waiver of co-pay (care at 100%). Therapeutic education is indispensable to optimize the care and compliance of the patient with the therapeutic project.

#### The essentials in 30 points

1. The diagnosis of Behçet’s disease is clinical. The pediatric and familial forms may be syndromic (of genetic origin).
2. HLA-B51 does not confirm or invalidate a diagnosis of Behçet’s disease.
3. A case of active Behçet’s disease in a young man calls for alert and careful monitoring. These patients are, in fact, at greater risk of severe complications.
4. The buccal aphthosis of Behçet’s disease is recurrent (> three attacks per year) and must be objectified by a physician. It does not systematically precede severe organ involvement (ocular, neurological, and/or cardiovascular) but physicians must systematically ask about it.
5. Genital aphthosis must be distinguished from other causes of genital ulceration and must be objectified by a physician along with a dermatologist or a gynecologist.
6. Perianal aphthosis is rare and must trigger elimination of an IBD (inflammatory bowel disease).
7. Articular involvement is most often oligoarticular and not destructive in Behçet’s disease. It readily affects the large joints and more rarely the spine. Axial damage may suggest spondyloarthropathy.
8. Ocular involvement is potentially serious and requires systematic seeking. Uveitis of Behçet’s disease is never granulomatous.
9. Every posterior uveitis in Behçet’s disease must be treated with systemic corticosteroid and immunosuppressant therapy, and severe cases (reduction of visual acuity, occlusive vascularity, and/or macular edema) justify the introduction of corticosteroids and anti-TNFα. Interferon-α is an alternative therapeutic agent.
10. Unexplained fever or biological inflammation in Behçet’s disease must trigger a search for a complicated/severe organ involvement such as cardiac and/or vascular .
11. In case of febrile headaches during the course of Behçet’s disease, cerebral thrombophlebitis must be ruled out by an angio-scanner or angio-MRI and then CSF examination to identify meningitis; parenchymal neurological involvement frequently parallels aseptic meningitis with lymphocyte predominance or neutrophils.
12. Cerebral thrombophlebitis justifies a search for intracranial hypertension. Optical atrophy and blindness may occur in case of persistent, overlooked intracranial hypertension.
13. In case of parenchymal damage, the cerebral lesions are preferentially located in the brainstem and central basal ganglia and at the capsulo-thalamic level.
14. In the presence of meningo-rhombencephalitis, infectious causes (listeriosis, tuberculosis) must be searched for carefully before imputation to Behçet’s disease.
15. Vascular involvement may be multiple and associates venous and arterial lesions, generally of large- and medium-sized vessels.
16. Superficial venous thromboses often precede deep venous ones.
17. Venous thromboses of Behçet’s disease are of inflammatory type. This justifies systemic administration of anti-inflammatory treatment with corticosteroids and, potentially, an immunosuppressant (azathioprine, cyclophosphamide) or an immunomodulator (anti-TNF-α), as well as an expert advice. The prescription of an anticoagulant is controversial, but may be considered in adults during the acute phase and in the absence of a risk of hemorrhage, particularly connected with associated arterial aneurysms.
18. The occurrence of venous thrombosis in children and young adults justifies a search for others causes of thrombophilia.
19. Severe organ damage (ocular, digestive, neurological, cardiovascular, and pulmonary) requires care by expert centers.
20. A multidisciplinary team is indispensable for the care of Behçet’s disease.
21. Ocular and neurological damage is the primary cause of sequelae and handicaps in Behçet’s disease.
22. Severe vascular manifestations (arterial pulmonary and aortic aneurysms and Budd-Chiari syndrome) are life-threatening.
23. Corticosteroid sparing is an essential objective, given the cortico-dependent character and frequency of relapses during the course of Behçet’s disease.
24. Any uncontrolled Behçet’s disease must prompt investigation into a lack of therapeutic compliance.
25. Colchicine is the first-line treatment of mucocutaneous and articular manifestations.
26. Colchicine (at the posology of 1–2 mg/day) must be continued for a minimum of 3–6 months before judging its ineffectiveness.
27. The posology of colchicine must be adapted to renal and hepatic function. Its prescription must involve precautions regarding drug interactions and grapefruit consumption. http://ansm.sante.fr/var/ansm_site/storage/original/application/a90a7e83a649086c46aa73ea1f9e1b56.pdf
28. Reduction (or cessation) of immunosuppressant or immunomodulating treatment can be considered, except in exceptional cases, only after at least 2 years of remission within the framework of severe Behçet’s disease (ophthalmological, digestive, neurological, cardiovascular, and pulmonary damage). Any treatment cessation requires expert advice.
29. Behçet’s disease is a chronic disease, which requires regular and extended follow-up associated with therapeutic education.
30. A transition from pediatric to adult medicine must be set up with adolescents affected by Behçet’s disease.

### Epidemiology

#### Behçet’s disease in adults

Behçet’s disease (BD) has significant epidemiological aspects to keep in mind, because they may have implications for the diagnosis. The most notable characteristic is the clear geographical disparity in the prevalence of BD. The countries and regions with the highest risk of BD are Turkey and the Asian (Japan, China, and Korea), Middle Eastern, and North African countries. Within Europe, BD is most frequent in Southern Europe, whereas the disease is very rare in Northern Europe. This is the same for the countries of North America. This specific geographical distribution has given BD the nickname the “Silk Road disease,” but this name is probably not appropriate, because the geographical distribution of BD is larger than this zone, since it ranges from Asia to the edges of the Mediterranean. Interestingly, this ethnic disparity persists in migrants (or descendants of migrants) who live in zones where BD is naturally rare, such as persons of North African or Turkish origin.

Another remarkable epidemiological characteristic of BD is the age of onset of the disease, which occurs relatively early in life. The average age at diagnosis of BD is approximately 30 years, and most diagnoses are made in patients between 15 and 45 years old. It is exceptional to make a new diagnosis of BD before the age of 15 years and after the age of 50 years. Men and women may be affected by BD, the sex ratio of which is close to 1 on the international scale. There are, however, some uncertainties concerning a possible greater gender-related predisposition to developing BD in certain geographical zones. Data giving the preferential occurrence of BD as a function of gender rely on the fact that gender is a determining factor in the severity of BD. Indeed, men present a more severe disease course, particularly a greater frequency of major vascular and ocular events. It is thus conceivable that the variation in sex ratio observed in the studies reflects a greater or lesser disposition to diagnose less severe forms of BD.

Several other elements highlight the important role of genetics in BD, the first of which is the well-established link between BD and the B*51 allele of Class I HLA. Approximately 30–60% of the patients carry this allele, while it is present in 10–20% in the general population. The geographical distribution of HLA-B*51 in the general population roughly overlaps with that of the frequency of BD. This provides a supplemental element in favor of the role of this allele in the pathogenesis of BD, knowing that it is neither necessary nor sufficient for the development of the disease and that it may explain only a part of the etiology. Apart from HLA-B*51, HLA alleles of class 1, such as A*26, B*15, and B*27, and of other genes, such as that coding interleukin 10, and pyrin (*MEFV*) have been identified as genetic factors related to susceptibility to BD. They have, however, a lower load than HLA-B*51. The frequency of the familial forms with several members suffering from BD demonstrates the influence of genetics on this disease.

Apart from genetic factors, environmental risk factors can influence the appearance of BD. In terms of infection, different bacterial or viral agents (such as streptococcus or the Herpes simplex virus) have been studied as etiological factors of BD.

#### Behçet’s disease in children

While particularly rare at the pediatric age, BD may occur at a median age of 8 years and with an equivalent distribution between the genders. BD is recognized on the average after 6 years of development in this age group, where the symptoms are not numerous and often appear in succession. The geographical factor remains preponderant, with a higher frequency of cases in the populations known to be at risk of BD. The incidence of pediatric BD (BDP) is not known, but these cases represent, according to the sources, between 3 and 26% of all cases of BD; they are distinguished by a high familial aggregation (10 times more frequently than in adults) underlying a more expressive genetic component. Boys experience more severe BD, with more ocular and vascular damage, while girls have more genital aphthoses. BDP cases occurring before the age of 8 years justify thorough searches for a Mendelian genetic cause.

#### Diagnosis, differential diagnoses, and evaluation of the activity of the disease

##### In adults

The diagnosis of BD is essentially clinical and is controversial. There are several classification criteria for BD, in fact designed toward clinical research rather than for making a definite diagnosis. Consequently, in current clinical practice, these different classifications are to be used with caution, since none allows identifying or ruling out BD with certainty. The most frequently used international classification criteria for BD are those of the International Criteria for the Classification of Behçet’s Disease revised in 2013 (Table 1).

**Table 1:** Behçet’s disease is diagnosed if ≥ 4 points are present

Symptoms	Points
Buccal aphthae	2
Genital aphthae	2
Ocular damage	2
Cutaneous damage	1
Vascular damage	1
Neurological damage	1
Positive pathergy test	1

Other elements may contribute to the diagnosis. In France (or in countries where “local” BD is rare), a diagnosis of BD seems more probable when it involves a person originating in a geographical region where BD is highly prevalent. Every case of serious organ damage may be isolated (e.g., thrombosis of the vena cava, sub-hepatic veins, pulmonary aneurysms, cerebral and retinal thromboses, etc.) and may bring about BD and require a systematic expert examination. A family history of BD also increases the diagnostic probability.

##### In children

The positive diagnosis of BD is exclusively clinically based and based upon evaluation. Diagnosis of BD is very difficult to establish at the pediatric age, because the symptoms are often not numerous and a significant number of differential diagnoses exist. Classification criteria adapted to children have recently been proposed (Table 2): recurrent buccal aphthosis, genital aphthosis, uveitis, cutaneous damage, neurological damage and vascular damage with a slightly modified description, and an absence of weighting. Children are classified with a BDP if they have a score ≥ 3.

**Table 2:** The provisional classification crieria for pediatric Behçet's disease

Item	Description	Value/item
Recurrent oral aphthosis	At least 3 episodes/year	1
Ulceration or genital aphthosis	Typical with scarring	1
Cutaneous damage	Necrotic folliculitis Acneiform lesions Erythema nodosum	1
Ocular damage	Anterior uveitis Posterior uveitis Retinal vasculitis	1
Neurological signs	With the exception of isolated headaches	1
Vascular signs	Venous thromboses Arterial thromboses Arterial aneurysms	1

In children, there is a growing list of differential diagnoses, of which the majority are of genetic origin (Appendix 2).

#### Physiopathology

Although the etiopathogenesis of BD remains unexplained, new data suggest that the inflammatory reaction during the course of BD results from a disturbance of the homeostasis of the innate and adaptive immune responses in genetically predisposed individuals. From this an activation of the T lymphocytes follows at the level of the peripheral blood and inflammatory sites. HLA-B*51 remains the key genetic susceptibility factor. Recent genomic studies have reinforced these data and made it possible to discover new susceptibility genes (IL-10, IL-23R, IL-12RB2). An infectious bacterial agent could be the trigger for the disease through an abnormal response of the T cells in relation to bacterial heat shock proteins (HSP) secondarily provoking, through cross-reactivity, the proliferation of autoreactive T cells in relation to human HSP.

Different infectious agents (*Streptococcus sanguis*, herpes simplex virus, tuberculosis, etc.) have been studied. Recently, an imbalance of the T lymphocytes consisting of the expansion of Th1 and Th17 and a reduction of the T lymphocyte regulators (Treg) has been demonstrated. Cytokines of type IL-17, IL-23, and IL-21 play a determining role. The most important cells involved in inflammation during the course of BD are the polynuclear neutrophils, NK cells, CD4 + T lymphocytes, and cytotoxic CD8 + T cells. Finally, endothelial cell dysfunction could play a role in BD. Chronic inflammation present in BD indicates an increased oxidative condition that induces platelet, leucocyte, and endothelial cell activation through the release of proinflammatory cytokines and chemokines. Neutrophils of patients with BD are hyperactivated and exhibit increased phagocytosis and superoxide production and NETs, which potentially contribute to clot formation.

## Objective of the National Diagnostic and Care Protocol

### Objective

The objective of this National Diagnostic and Care Protocol (NDCP) is to explain the diagnostic care, current optimal therapy, and course of care to professionals in charge of patients with BD. The goal is to optimize and harmonize the care for and follow-up of this rare disease over the entirety of the area. It also makes possible the identification of the pharmaceutical specialties that are used in an indication, but which are not stated in the marketing authorization (AMM), as well as the specialties, products, or services necessary for the care of patients that are not usually provided or reimbursed.

This NDCP may serve as a reference for the general physician in cooperation with other specialized physicians.

The NDCP may not, however, consider all specific cases, comorbidities or complications, therapeutic aspects, hospital care protocols, etc. It cannot, therefore, claim to exhaust all possible care channels or be a substitute for the individual responsibility of the physician in relation to their patient. However, the NDCP described the current reference care of a patient affected by BD. It must be updated based on new validated data.

All the drug treatments are prescribed off-label during the course of Behçet’s disease.

### Work method

The present NDCP has been drawn up according to the “Method of Drafting of a National Diagnostic and Care Protocol for Rare Diseases” published by the Supreme Health Authority in 2012 (methodological guide available on the HAS site: www.has-sante.fr).

## Initial diagnosis and evaluation

### Objectives

- Learn to recognize symptoms suggesting a case of BD;
- Learn the means to confirm the diagnosis of BD and exclude differential diagnoses;
- Research and anticipate the possible complications of BD;
- Draw up a pre-therapeutic assessment.

### Professionals involved (and modalities of coordination)

Because of the extreme polymorphism of the disease, all physicians may be confronted with an incipient BD. The initial care of the patient affected by BD could, therefore, be ensured by:

- The general physician or a community pediatrician. The diagnosis must then be confirmed by a physician having experience with BD;
- The adult and pediatric specialists most frequently involved: internal medicine specialist, rheumatologist, ophthalmologist, dermatologist, neurologist, vascular physician, and gastroenterologist;
- Any other specialist whose opinion is necessary based on the clinical presentation may be consulted.

Expert advice and examination could be provided at reference centers, competence centers, and their networks of correspondents.

### Clinical examination

Since BD is a proteiform disease, the questioning and physical examination are the key steps for the diagnosis and must be exhaustive.

BD develops by unexpected flares, without strict parallelism between the mucocutaneous and visceral lesions. Fever is rare in the non-complicated forms of BD; its presence must then trigger the search for an underlying vascular complication.

#### Dermatological manifestations

Mucocutaneous manifestations need to be determined. Their presence is a crucial help for a definitive diagnosis, three of the four classification criteria of BD being of a dermatological nature. These manifestations may precede or occur concomitantly with the other systemic manifestations. Apparently common, they are to be systematically sought out during the questioning of the patient. They may sometimes occur several months, even several years after the other manifestations. Their absence makes a definitive diagnosis difficult, explaining important diagnostic delays.

**Buccal aphthae** are present in 98% of the cases. This involves painful single or multiple ulcerations ranging from a few millimeters to > 1 cm in diameter with sharp edges, sometimes preceded by an ephemeral vesicle. The ulceration is lined by a “fresh butter” coating or grayish appearance, and its edge is inflammatory. The buccal aphthae must be identified by a physician to eliminate the numerous other causes of recurrent buccal ulcerations (traumatic ulcerations, polymorphic erythema, etc.). They are found on the internal surface of the lips, cheeks, gingivolabial groove, edge of the tongue, frenulum, buccal floor, palate, tonsils, and pharynx. Typically unrestrained, they may also be promoted by food (skin of fruits, nuts, hazelnuts, almonds), dental trauma, and sometimes menstrual cycles and emotions (stress). When they are numerous or large, they may disrupt eating and speech. The development proceeds spontaneously toward healing without scarring, and they are not accompanied by adenopathy. They may not in practice be differentiated from idiopathic buccal aphthosis, but their number, repetition, and debilitation that they cause must direct the physician toward BD.

**Genital aphthae** exist in 60–65% of the cases and are suggestive of BD when they are seen, whether during the acute or secondary scarring phase. The genital aphthae are often deeper and bigger than buccal aphthae. They recur less often than the latter but the depigmented scars allow a retrospective diagnosis. In men, they are located on the scrotum or rarely on the penis or in the urethra; in women, they are found on the vulva, vagina, and uterine cervix. They may be disseminated and painful or entirely latent.

The aphthae may be located on the esophagus, stomach, intestine (exceptionally entailing perforations), and anal margin. Large, rounded cutaneous ulcerations, semiologically similar to aphthae, that most often preferentially multiply on the internal surface of the thighs are also described.

Among other cutaneous manifestations, papulo-pustular lesions not centered on a hair (pseudo-folliculitis, acneiform lesions) and cutaneous hyperreactivity not specific to epithelial damage (site of injection, infusion, a superficial scratch, or intradermal reaction with various antigens) are the most characteristic. Hyperreactivity is the basis for the pathergy test. This test is considered positive when a papule or pustule is caused 24–48 h after intradermic pricking at 45° in relation to the anterior face of the forearm by a 20–26 G needle. The sensitivity of this test is reduced by the use of disposable material and cutaneous disinfection.

Some hypodermic nodules are often found; these involve either:

- A readily recurrent erythema nodosum reported in approximately 1/3 of the subjects or
- Superficial thrombophlebitis presenting in the form of erythematous nodules sometimes in linear arrangement, often confused with erythema nodosum.

Other cutaneous manifestations are described, such as purpura and necrotic lesions, in connection with the cutaneous vascularity and lesions of neutrophilic dermatosis.

#### Articular and muscular manifestations

**Articular involvement** is early, sometimes initiatory, and can precede the other manifestations by several years. It involves arthralgia and/or, rarely, “inflammatory oligoarthritis” that is generally fixed, localized at the level of the supporting joints (knees, ankles). Damage to the small joints of the hands and feet is rare. Temporo-maxillary, sterno-clavicular, manubrio-sternal, and atloido-axoidian damage and damage to the hip are exceptional. Development is recurrent and asymmetrical. Polyarticular forms are rare (2%). X-rays are generally normal; osteocartilaginous erosions or minimum pinchings may exceptionally be found. Articular destruction is exceptional. Deformation may then be observed. An articular puncture reveals a viscous, inflammatory liquid, rich in polynuclear materials.

Occurrence of popliteus cysts is possible, and their rupture may be difficult to differentiate from a thrombophlebitis, especially since associations have been observed. According to the authors, variable damage of the sacroiliac is found (1–34%), as is association with authentic ankylosing spondyloarthritis in HLA-B27 subjects (2%). Some osteonecrosis has been reported, generally in patients treated with corticosteroids.

**Muscular involvement** is rare but indisputable and may be associated with articular manifestations. It is essentially expressed by diffuse myalgias or predominantly in the proximal muscles, and true myositis is possible. Localized forms may pose problems for the differential diagnosis with a thrombophlebitis. Painful tumefactions may be noted during the examination. The biopsy shows a degeneration of muscular fibers and infiltration by mono- and polynuclear cells. The CPK levels are exceptionally elevated, and when they are elevated, myopathies and the rare colchicine-related secondary rhabdomyolysis reported in the event of renal or hepatic insufficiency need to be considered.

#### Ophthalmological manifestations

**Ocular manifestations** are third in frequency after cutaneous and articular ones. They affect the functional prognosis, especially since the bilateralization of the lesions may be rapid (2 years on average). They are characterized by recurrent flares of intraocular inflammation associated with a risk of blindness.

Both the anterior and/or posterior segments of the eye may be affected.

*Anterior uveitis* is a non-granulomatous uveitis. Sometimes quiescent, it may be visible only under a slit lamp during the examination. Subjectively assessed by the Tyndall rating, it may be objectively measured by a laser flare meter. This, in particular, exposes crystalline synechiae and hypertonia. It may be accompanied by a hypopyon, which is suggestive of BD. Anterior uveitis is rarely isolated (10% of cases) but is usually associated with damage to the posterior segment.

In fact, in case of ocular damage, posterior uveitis is practically constant. The damage may be paucisymptomatic, and any BD justifies a systematic ophthalmological examination. Damage to the intermediary and posterior segment may uncover hyalitis, retinal vasculitis that is essentially venous and often occlusive, macular edema, and foci of retinal necrosis. Young age, male sex, and the severity of the initial damage are associated with poor visual prognosis. Retinal vasculitis may be occlusive and necrotizing, associated with a vitreous Tyndall effect. These vasculitides are marked by a whitish edematous perivenous, then periarterial sheathing visible at the base of the eye, sometimes solely on the periphery or seen by fluorescein angiography, which shows the capillary dilatations with zones of obstruction and leaks. The occlusive character of the lesions is marked by hemorrhages and retinal edema and may lead to blindness. The extension of an ischemia may be complicated by neovascular pre-retinal proliferation. Macular edema is the most feared complication, which leads to central blindness if it is ischemic. The vitreous humor is secondarily affected, loses its transparency, retracts, and hardens, having a tendency to pull on the retina, which it may tear. Other ocular lesions are rarely observed: conjunctival aphtha, episcleritis, and keratitis.

The prognosis of these ocular lesions is severe. The lesions develop by flares. They may lead to major complications such as cataracts, ocular hypertonia, and blindness through damage to the posterior segment.

Previous studies have shown that one patient in two might be blind within 5 years following the first ocular symptom. Currently, under the cover of good therapeutic observance and care adapted by specialized teams knowing how to use corticosteroid therapy and immunosuppressants and to organize regular follow-ups, the incidence of blindness has been reduced.

Ocular damage may also be integrated into neurological lesions: optical neuropathy, paralyses of the motor nerves of the eye, papillary edema with intracranial hypertension by intracerebral venous thrombosis, papillitis always entailing a reduction of the visual acuity, and damage to the visual field.

#### Neurological manifestations

The neurological manifestations of BD are essentially connected to damage to the central nervous system of the “parenchymal” type (damage of the white substance, preferentially of the meso-diencephalic junction) or “extra parenchymal” (venous or arterial vascular damage). Peripheral nervous system involvement has rarely been described, but its connection with BD remains disputed.

Neurological manifestations occur in approximately 25–30% of the patients. Parenchymal involvement is the most frequent (70–80% of patients with neurological damage). The coexistence of both parenchymal and extraparenchymal involvement in the same patient is exceptional. Neurological involvement may be initiatory of BD, especially before the mucocutaneous manifestations appear or are diagnosed (Table 3).

**Table 3:** Diagnosis of neuro-Behçet’s disease according to an international consensus in 2014

1. Neuro-Behçet’s disease definition:
A. Patients responding to the international criteria for the diagnosis of BD
B. Clinically identified neurological syndrome in connection with Behçet’s disease and associated with anomalies of neuroimaging and/or of the CSF analysis
C. No etiological alternative to the symptoms presented
2. Probable neuro-Behçet disease:
A. Objective neurological syndrome as defined in a patient meeting one but not all of the diagnostic criteria of BD
B. Non-characteristic neurological syndrome in a patient with defined BD

##### Clinical presentation of neurological parenchymal involvement

In clinical terms, the most representative symptoms are: headaches (> 50% of cases), associated with a pyramidal uni- or bilateral syndrome (50–90%), a cerebellar ataxia, and sphincter disorders (25–40%). This diffuse involvement of the cerebral parenchyma may also take the form of encephalitis with psychomotor slowing with behavioral disorders being the most prominent sign. Epileptic manifestations occur in 5–10% of the cases. Lymphocytic meningitis or an aseptic type may occur.

Involvement of the cranial nerves, cerebellar syndrome, sensitive disorders, abnormal movements, or extrapyramidal syndrome may also be observed. Isolated headaches are a frequent symptom during the course of BD. Their modification must trigger the search for anomalies on the neurological examination, cerebral venous thrombosis, and associated meningitis or uveitis.

Isolated medullary damage is rare and mostly of the transverse myelitis type. It may reach all of the medullary segments.

In two-thirds of cases, the neurological manifestations are acute and develop by crises interspersed with periods of remission (with or without sequelae of previous outbreaks). Nevertheless, progressive primary or secondarily progressive forms may be found.

##### Clinical presentation of neurological extraparenchymal involvement

Neurological extraparenchymatous involvement is essentially represented by cerebral thrombophlebitis.

In cases of cerebral venous thrombosis, the clinical signs are essentially symptoms of intracranial hypertension (most particularly headaches and papillary edema), fever, and, more rarely, a focal deficiency or comitial crisis. All the veins may be damaged but thromboses of the superior sagittal sinus and transverse sinus are the most frequent. The manner of revelation of the symptoms may be sudden (< 48 h) or progressive (< 1 month).

Damage to arteries with cerebral targets are much rarer. Essentially occlusions of the carotid and vertebral arteries are found, as well as aneurysms with a risk of hemorrhagic rupture.

#### Vascular manifestations

The very specific damage of BD at the level of the vessels can only suggest the diagnosis of BD. The vascular damage causes no authenticated thrombophilic abnormalities of hemostasis.

**Venous thromboses**

They occur in almost 30% of cases. Superficial venous thromboses are transitory and migratory and found during questioning and clinical examination.

**Deep venous thromboses**

They may affect all the axes, are often recurrent, and are sometimes indicative. It is necessary to be particularly attentive in the presence of certain proximal or atypical locations: ilio-femoral, vena cava, sub-hepatic, and cerebral veins. BD must be considered in the presence of unexplained thrombosis in a young adult, without other vascular risk factors and with an assessment of negative thrombophilia. These thromboses often accompany an inflammatory biological syndrome. Venous involvement may be multiple and associated with arterial involvement.

Hughes–Stovin syndrome (association of deep vein thrombosis with arterial pulmonary aneurysms) includes a high risk of hemoptyses.

The intracranial venous thromboses may be complicated by blindness, because of the papillary edema that must be systematically sought.

**Arterial involvement**

They are currently better recognized and observed in 5–10% of cases, depending on the series. This frequency is probably underestimated if one considers the autopsy data, in which arterial damage was observed in one in three patients. This may involve occlusions or arterial aneurysms (genuine “arterial aphthae”) with a risk of rupture, often multiple, localized on the pulmonary vessels, the aorta, and peripheral arteries (usually in the lower limbs than in the upper limbs). All arteries may be affected. Patients may be asymptomatic if collaterals secondary to an occlusion develop or they present signs of acute ischemia in case of acute arterial thrombosis, or hemoptyses, thoracic/abdominal pain, or a life-threatening pattern in the event of the rupture of a pulmonary or arterial aneurysm in another location.

Aneurysms may occur at the sites of arterial puncture, so non-traumatic techniques are favored.

Aneurysmal pulmonary artery damage is rare, serious, and marked by sometimes massive hemoptysis. The extremely severe prognosis (mortality estimated at 60%) has been improved because of better diagnosis of this complication, the introduction of medical treatments with the systematic use of immunosuppressants, and use of interventional radiology. The indication of anticoagulation in the event of associated thrombosis must be adapted on a case-by-case basis, particularly after examination by an expert center.

The prognosis for arterial damage is poor, since it is the primary cause of death (approximately 30–40%).

**Cardiac involvement**

They (< 5% of the cases) may involve all three entities: myocarditis, which can manifest under rhythm disorders, endocarditis with aortic or mitral valve disease, and fibroblastic endocarditis sometimes complicated by intra-cavitary thrombi. Pericarditis can be inaugural and readily recurrent. Coronary damage is also described with aneurysms and thromboses complicated by myocardial infarction, hemopericardium, and/or sudden death.

#### Digestive manifestations

Macroscopically, gastrointestinal manifestations resemble Crohn’s disease lesions, and more rarely those of hemorrhagic rectocolitis (diffuse damage restricted to the rectum and colon), then posing nosological difficulties. Thus, the frequency is variously assessed, ranging from 30% in the Japanese series to < 5% in the European series.

The functional symptomatology is not specific: flatulence, nausea, bloating, belching, diarrhea, anorexia, or rectal bleeding. Neither specific endoscopic nor histological aspects are reliable; however, the depth of the lesions, which are often not numerous (< 5), with oval shape and ileocecal location, indicates BD. Unlike in Crohn’s disease, granuloma is never noted in the biopsies. A volcano-shaped ulcer in the ileocecal region is suggestive of BD. The histology frequently reveals non-specific inflammatory lesions. Some cases of pancreatitis have been reported.

A digestive presentation as the most prominent sign must trigger the search for a differential diagnosis (CIID) or a genetic cause (see the chapter on requesting a genetic diagnosis).

#### Pulmonary involvement

Pulmonary involvement is rare and essentially consists of nodular lesions of post-ischemic pulmonary consolidation, which then have a tendency to expand. Infiltrates, such as frosted glass, indicating an intra-alveolar hemorrhage, may sometimes be observed. In some cases, vascularity is detected by angio-TDM and ventilation/perfusion scintigraphy.

#### Renal involvement

Renal involvement is exceptional and is dominated by amyloid nephropathy, which always occurs with sick patients not monitored after numerous years of development. Some rare observations report glomerular nephropathies dominated by proliferative glomerulopathies with increasing epithelial and glomerular nephropathies to deposits of IgA. Complications connected with thromboses of the renal veins and/or arteries have been reported.

#### Genital involvement and fertility

Testicular or epididymis damage: orchitis and/or orchiepididymitis has been reported by various authors.

**Fertility**

Fertility does not appear modified during the course of BD, by either the disease itself or colchicine treatment. BD may affect young women of reproductive age. The incidence of flares of BD decreases during pregnancy and post-partum, particularly in female patients treated with colchicine. Thus, taking colchicine appears to be effective in reducing the risk of BD outbreaks without additional danger to the fetus. The complications of pregnancy are not increased during the course of BD. Pregnancy cannot be recommended in case of severe organ damage involving the vital and/or functional prognosis and requiring potentially teratogenic immunosuppressive treatment. There are anecdotal cases of neonatal Behçet’s disease with cutaneous lesions, usually with a transient evolution.

#### Behçet’s disease in children

##### Clinical

The clinical signs of BDP may involve numerous organs and are not very different from those of adults; they often appear in succession, or more rarely simultaneously, between the ages of 8 and 11 years, and this is most often recurrent buccal aphthosis, which indicates the beginning of BD (data from the larger international cohort of BDP: PEDBD, 219 patients analyzed).

##### Fever

Although rarely observed in non-complicated BD in adults, recurrent fevers are present in 44% in patients of the PEDBD cohort. These fevers may be accompanied by outbreaks of buccal aphthosis at the beginning of the disease and pass for PFAPA syndrome. During the course of BDP, a fever associated with an elevation of proteins in the acute phase of the inflammation (CRP) may be indicative of severe neurological or vascular damage and must, therefore, be an impetus for investigation. The presence of recurrent fever must also lead to differential diagnoses of hereditary autoinflammatory syndromes.

##### Mucocutaneous manifestations

In children, the buccal aphthae are, in 80% of cases, the first manifestation of BDP and occur at an average age of 7.4 years. The recurrence of at least three episodes per year is a key element of the diagnosis, and the lesions must be assessed by a physician. In the international PEDBD cohort, on average, children had 12 episodes of buccal aphthae per year.

Genital aphthae are rare before puberty and most frequently affect young girls (96%). Their flares are on average four per year. Perianal ulcerations are more frequent in children (7% of cases) than in adults and raise the problem of the differential diagnosis with inflammatory bowel diseases. Cutaneous damage often begins around the time of puberty and is dominated by pustular lesions (40–60%) and erythema nodosum (40%). Pseudo-folliculitis is essentially located over the buttocks and at the base of the thighs. It is more frequent in boys and is present in the form of erythematous papules topped by a pustule not centered on a hair and covered by a blackish crust within 24–48 h. Pustular lesions on the face may be confused with common acne vulgaris.

##### Articular manifestations

Arthritis is present in approximately 30% of patients. However, articular involvement is more frequent when isolated arthralgia is taken into account (50% in the PEDBD cohort). Articular manifestations usually affect the large joints like the knees and ankles and are, generally, neither erosive nor deforming. They develop by flares and may be accompanied by transient aggravation of aphthosis and erythema nodosum. Axial involvement (17%) and/or enthesopathy can occur in patients bearing the HLA B27 antigen (2%). Peripheral damage involves 48% of the cases.

##### Ocular manifestations

Ocular damage appears, on average, at the age of 11 years in the PEDBD cohort. Anterior involvement includes iridocyclitis with red eye, which is painful, and photophobia. Posterior involvement may be more insidious in young children, being manifested by visual blurring, metamorphopsia (distorted vision), or myodesopsia (flickering), which delay the diagnosis and aggravate the prognosis. Uveitis is remarkable for its recurrent character and for its severity, leading to blindness in 10% of cases. Anterior uveitis (rarely isolated) is seen in 24% of cases and posterior uveitis in 21% of cases. The most characteristic feature is panuveitis (anterior and posterior), which is accompanied by a puriform effusion of the anterior chamber in 19% of cases that may manifest as hypopyon. Ocular involvement is most often bilateral and nearly always includes retinal vasculitis, which manifests as retinal vascular hemorrhages and thromboses (venous 10%, arterial 2%). Other ocular manifestations, including papillary edema (8%), papillitis, and retrobulbar optical neuritis, arise directly from the cerebral vascularity. Boys clearly have more frequent ocular involvement than girls do and present, above all, posterior uveitis, retinal vasculitis, papillary edema, and venous thrombosis.

##### Vascular manifestations

Superficial and deep venous thromboses characterize BD and are present in approximately 10% of patients, primarily at the lower limbs. Arterial damage, which is less frequent (2–5%), almost always is indicated by its complications, particularly the ruptures of aneurysms.

**Venous involvement**

BD is the primary non-neoplastic disease that can lead to thromboses in the child. However, embolic complications are exceptional (< 1%). Deep venous thromboses may involve the vena cava, femoro-iliac, and cerebral veins. Damage of the superficial vessels is manifested by “subcutaneous” nodules and/or thromboses of the limbs. Thromboses are often multiple and recurrent in the same patient, with the presence of a collateral network.

**Arterial involvement**

Arterial involvement is the primary cause of mortality during the course of BDP. It is preferentially centered at the level of the pulmonary arteries and abdominal aorta. Lesions may be multiple and include thromboses and aneurysms simultaneously.

##### Digestive manifestations

These occur in 40% of cases of BDP and correspond, above all, to isolated abdominal pain or digestive discomfort. Ulcerative mucosa of Behçet may reach the entirety of the digestive tract and buccal cavity (4.5%). Colonic involvement is the most frequent (cecum, ascending colon) and manifests as abdominal pain with bloody diarrhea (2.5%). It may cause hemorrhages or perforations. Its presence absolutely must trigger the search for a disease other than BD: mevalonate kinase deficiency, A20 haploinsufficiency, IL-10/IL10R deficiency, or anomaly of chromosome 8.

##### Neurological manifestations and psychiatrics

Neurological manifestations are present in approximately 20–30% of the cases. A male predominance has been noted in several series. Neurological damage may sometimes be initiatory (10–33%) but, in most cases, they occur on average 2–5 years after the beginning of the disease. Neurological manifestations are non-specific, being most often expressed as headaches (92%), damage to the cranial pairs (38%), and sometimes convulsive crises (12%). Cognitive disorders, a pseudo-bulbar syndrome, or cerebellar disorders, which half the time are associated with various neuropsychiatric disorders (confused states, depressive syndrome, psychotic manifestations), have also been observed. Patterns of acute transverse myelitis have been described. Meningeal damage is frequent; this includes aseptic meningitis. It remains latent in more than one out of two patients, being expressed by isolated headaches. A pattern of “benign” HTIC is present in 20% of cases. It is expressed by frontal headaches associated with vomiting and paralysis of the VI with occasional meningeal stiffness. In most cases, cerebral imaging reveals, unlike in adults, a cerebral thrombophlebitis (89% in the Turkish series of Uluduz), which, if it is initiatory and isolated, must arouse suspicion of neuro-Behçet’s disease, with parenchymatous damage affecting the brainstem and particularly the diencephalon. Spontaneous development of neuro-Behçet’s disease moves toward progression of symptoms and multiplication of lesions. The prognosis for neuro-Behçet’s disease is bleak. The mortality is 5%, and serious sequelae are present in 80% of surviving patients. Fifty percent of children affected by neuro-Behçet’s disease may not follow a normal academic course, and 50% of them require special arrangements to remain in the school system.

##### Various manifestations

**Cardiac involvement**

Cardiac involvement is rare in children and is a result of the vascularity of the large vessels: aortic aneurysms, coronaries, and myocardial ischemia. Pericarditis and myocarditis with intracardiac thrombus are also possible.

**Pulmonary involvement**

Vascularity is essentially expressed by aneurysms of the pulmonary arteries, the rupture of which may be fatal. BPD can also be associated with parenchymatous lesions, such as nodules, cavities, pleural effusions, or mediastinal adenopathies. It is necessary to eliminate the infection in this context.

**Genito-urinary manifestations**

These are very rare and include, beside scrotal or labial ulceration, aseptic urethritis, and epididymitis.

**AA amyloidosis**

AA amyloidosis is a rare complication of BD. It may be suspected in the presence of a nephrotic syndrome or be discovered by chance during an autopsy. Amyloid deposits may involve the lungs, kidneys, thyroid, spleen, and adrenal glands. Amyloidosis is extremely rare in the early care of BDP.

### Confirmation of the diagnosis

#### Making the diagnosis

##### In the adult

The diagnosis of BD is clinical and based on a number of hints; classification criteria may help even though they cannot rule out a diagnosis of BD.

It is necessary to know how to make the diagnosis of BD in the presence of the suggestive clinical-radiological presentations (cardiovascular, neurological, ocular, etc.), even in the absence of all of these criteria, to prevent delays in care.

In the presence of clinical suspicion of BD, no additional biological criterion exists. Haplotyping of Class I HLA must not, therefore, be carried out. The biological inflammatory syndrome is irregular and more readily found in BD with vascular damage and/or in cases of arthritis.

Cutaneous biopsy of an intradermal reaction to the physiological serum sometimes makes it possible to observe a vascularity. However, this is not a diagnostic test.

BD is suggested in the presence of uveitis (posterior damage with occlusive vascularity and/or previous damage with hypopyon), uveo-meningitis and/or meningoencephalitis, a thrombosis of unusual location (Budd-Chiari syndrome, particularly when there is damage to the vena cava), particularly in the event of a severe and/or recurrent form in a young patient, and, a fortiori, if it originates in a highly endemic zone or in the event of a pulmonary aneurysm, false aneurysm, and/or recurrent thromboembolic disease, even without meeting all of the international classification criteria.

Suspicion of BD must trigger a search for the classification criteria, particularly the mucocutaneous signs (buccal and genital aphthosis, including scrotal scarring, erythema nodosum, pseudo-folliculitis) and/or asymptomatic ophthalmological anomalies providing a strong argument for retrospective diagnosis.

There is no standard for paraclinical explorations of BD, but the assessment must include a methodical and complete ophthalmological examination and venous and/or arterial Doppler imaging in the event of manifestations causing vascular damage. A cerebral MRI and/or LP will be systematic in the presence of persistent headaches and/or appearance of neurological symptoms. It is too early to determine the role of TEP scans in the screening and monitoring of visceral damage, particularly vascular.

**Differential diagnoses of BD**

The solely clinical character of the diagnosis involves the elimination of all differential diagnoses as a function of the presentation (Table 4).

**Table 4:** Differential diagnoses of Behçet diseases according to organ involvement

Type of damage	Differential diagnoses
Mucous ulcerations	Herpes, neutropenia, pemphigus, CIID, drugs, vitamin deficiencies
Bipolar aphthosis	CIID, MAGIC syndrome, deficiency of mevalonate kinase, A20 haplo-insufficiency
Articular damage	Spondyloarthropathies
Gastrointestinal damage	CIID, NSAID toxicity, infectious colitis
Venous thrombosis	Thrombophilia, genetic or acquired
Arterial damage	Septic aneurysms, atrophic polychondritis, Takayasu
Neurological damage	SEP, sarcoidosis, tumoral pathologies, lymphomas or infectious meningo-encephalitis

In particular, buccal aphthosis may commonly be found in the general population; on the other hand, bipolar aphthosis is clearly more suggestive of BD. The aphthae must be identified by a physician to eliminate other causes of mucous ulcerations (herpes, pemphigus, traumatic, severe neutropenia, etc.). Other causes of mucous ulcerations or aphthae may be found in Crohn’s disease, neutrophilic dermatoses, other autoinflammatory diseases, vitamin deficiencies, and when taking certain drugs (nicorandil, NSAIDs, sirolimus, etc.). For bipolar aphthosis, it is necessary to suggest a CIID, mevalonate kinase deficiency, A20 haplo-insufficiency, and MAGIC syndrome (Table 4).

Uveitis may lead to considering many etiologies for which an ophthalmological evaluation is indispensable. Venous damage must trigger the search for other situations of thrombophilia, whether genetic or acquired. These may sometimes be related to their specific frequency in the general population.

Arterial involvement must eliminate the presence of infectious arteritis and Takayasu disease. Polychondritis poses particular problems in this regard because of forms sharing the semiology of the two disorders (MAGIC syndrome for “mouth and genital ulcers with inflamed cartilage” syndrome).

Finally, the differential diagnoses of the neurological forms are: multiple sclerosis, sarcoidosis, tumoral pathologies, lymphomas, and infectious meningo-encephalitis.

##### Peculiarities of a diagnosis of BD in children

**Diagnosis**

The diagnosis of BDP is also a clinical diagnosis.

Three signs out of six are necessary to classify the patient as affected by BDP.

Since this involves only classification criteria, a patient not meeting these criteria may still have a BDP. This classification allows making the diagnosis of BDP with a sensitivity of 77% and specificity of 88%.

A study in 2017 showed that, out of 68 patients affected by BDP vs. 90 control subjects, these pediatric criteria were more sensitive (74% vs. 23%) and also more specific than the ISG criteria (usually used in adults) for making the diagnosis of BDP.

**Differential diagnosis of BDP**

The differential diagnoses are numerous and will be addressed as a function of the clinical presentation.

In the presence of a “true” and recurrent buccal and/or genital aphthosis, the following may be considered: idiopathic recurrent benign buccal aphthosis, inflammatory bowel disease (IBD), Mendelian autoinflammatory syndromes (HA20, mevalonate kinase deficiency MKD), and Behçet-like syndromes. In the presence of non-identified buccal ulcerations, an examination by a dermatologist is appropriate to eliminate herpetic infections in particular (the lesions are erosive and vesicular), justifying a smear to search for the virus and a PCR analysis, and bullous diseases damaging the buccal cavity (pemphigoid, pemphigus vulgaris, superficial pemphigoid requiring a biopsy) should be considered.

In the presence of gastrointestinal manifestations as the most prominent and severe sign (anorexia, nausea, vomiting, diarrhea, abdominal pain, deviation from the morphometric curve), it is necessary to eliminate the presence of an IBD (primarily Crohn’s disease) or any other cause of ulcerative colitis, including deficiencies of IL10 and IL10R, MKD, and HA20 and anomalies of chromosome 8.

Haplo-insufficiency of A20 has bipolar aphthosis in common with BD, which is severe and sometimes destructive, and uveitis, which has previously been reported in most cases. Inflammatory flares are accompanied by a clear elevation of the CRP. Genetic transmission of this disease is dominant. Compared with BD, the beginning of symptoms is earlier, and severe digestive damage is the most prominent sign. Patients may, during the course of their lives, present autoimmune manifestations (lupus; association of dysimmunity of the type diabetes, autoimmune cytopenia, IBD, hepatitis, and interstitial pneumopathy; autoimmune lymphoproliferative syndrome, ALPS) and sometimes a discreet humoral immune deficiency, and febrile episodes are more consistent than in BD.

In the presence of recurrent fever, the Mendelian autoinflammatory syndromes (FMF, TRAPS, MKD), multi-factorial syndromes, such as PFAPA, constitutional pathologies of the neutrophils (cyclical neutropenia, myelodysplasias, septic granulomatosis), and deficiencies of the adaptive immunity must be considered.

In case of a neurological presentation (meningitis and/or encephalomyelitis), the main differential diagnoses are multiple sclerosis, sarcoidosis, tuberculosis, and lymphoma. Isolated cerebral venous thromboses require the search for an infectious cause, systemic lupus erythematous, and a procoagulant state (primary syndrome of antiphospholipids, protein S or C deficiency, and homocystinuria).

##### Role of genetics

A genetic cause is highly suspected if there is:

- early onset of symptoms
- family history
- unexplained recurrent fever associated with elevated CRP
- digestive symptoms (most prominent)
- dysmorphia
- myelodysplasia.

An expert center must be found in these cases (Appendix 1).

Genetic causes must be suspected in the presence of very early onset (first years of life), a fortiori, outside the groups with habitual risks, family history, a pronounced autoinflammatory phenotype (recurrent fever and biological inflammation), frequent gastrointestinal damage, sometimes severe, dysmorphic features, and/or atypias in relation to the classic phenotype of BD. Besides these identified genetic causes, pediatric BD must be distinguished from PFAPA syndrome (periodic fever, adenitis, pharyngitis, and aphthosis), an essentially pediatric entity that includes regular outbreaks of elevated fever, over 3–7 days, accompanied by a general malaise with headaches and myalgias, exudative pharyngitis, and often sensitive cervical adenitis. Buccal aphthosis is generally very moderate and irregular. This entity, which regresses spontaneously over time, has a transitory nature; it may constitute a mode of entry to a more characteristic systemic disease, of which BD forms a part.

## Therapeutic care

### Objective

- In the short term:
- Provide daily comfort;
- Sometimes ensure functional or even vital rescue;
- Screen for potentially serious damage.
- In the medium term:
  Counteract the foreseeable development of serious damage, particularly ophthalmological, neurological, and vascular;
  Prevent outbreaks;
  Prevent thrombotic manifestations;
  Maintain quality of life and socio-professional integration;
  In children:
  Maintain schooling and academic education;
  Maintain psychosocial development of the adolescent;
  Monitor stature and pubertal development and discusses specific treatment in cases of abnormalities;
  Ensure transition of care from pediatrics to adult medicine.
- In the long term:
  - Limit disease sequelae;
  - Limit the different detrimental effects of the treatment.

### Professionals involved (and modalities of coordination)

Treatment is ideally coordinated by a pediatrician or adult practice physician having expertise in BD, most often an internal medicine specialist, dermatologist, or rheumatologist. The general physician or pediatrician optimizes the coordination of the proposed care plan. Other specialized physicians may complete the patient care: ophthalmologists, neurologists, vascular physicians, gastroenterologists, and pulmonologists. The role of general physicians and pediatricians is essential in the first-line evaluation of the patient. Other health professionals may be called upon as necessary: professionals in therapeutic education, dietitians, state-certified nurses, physiotherapists, work therapists, psychologists, and social workers.

### Therapeutic care

#### General measures

Corticosteroid therapy increases the risk of infection and may favor the emergence of latent infections. It is thus appropriate to prevent them by vaccination and anti-infectious prophylactic treatments when necessary. Seasonal vaccination against the flu should be proposed to patients with BD. The anti-pneumococcal vaccination is also recommended. Vaccination against pneumococal infections must be carried out with the 13-valent conjugated polyosidic vaccine in accordance with the scheme adapted to the patient’s age and follow-up administration of the 23-valent polyosidic non-conjugated vaccine (if age > 2 years).

Vaccines recommended for patients receiving immunosuppressants, biotherapy, and/or corticosteroid therapy for an autoimmune or chronic inflammatory disease are those of the vaccination calendar that is in effect. Vaccination against papillomavirus should be considered in adolescents, even from the age of 9 years.

It is recommended to update vaccinations as soon as possible during the course of the disease, and before the initiation of immuno-suppressor treatment if possible, particularly for live-attenuated vaccines, which could then no longer be administered.

Recent contact with a person suffering from tuberculosis, with a previous history of tuberculosis not treated and spontaneously healed, an intradermal reaction to tuberculin > 10 mm in the absence of vaccination by the BCG, or a positive Quantiveeron^®^ test must lead to consideration of anti-tuberculous prophylaxis together with introduction of corticosteroid therapy. In cases of prescription of rifampicin, the corticosteroid therapy doses must be increased by approximately 30% to counteract the effect of enzymatic induction of rifampicin.

Strongyloidiasis hyperinfestation or malignant strongyloidiasis must be prevented by an anti-parasitic eradication treatment at the time of introduction of corticosteroid therapy in all patients who have stayed in an endemic zone (tropical or subtropical regions, Southern Europe).

Prevention of secondary metabolic effects of a corticosteroid therapy prescribed in the long term (other than osteoporosis) represents another major factor in the prescription of corticosteroids. In children, long-term steroid doses > 0.3 mg/kg impair both height and weight velocity and induce pubertal delay. In teenagers, physical changes in appearance (facio-truncal obesity, hyperpilosity, and striae) are unbearable and frequently cause lack of compliance with treatment. The early use of immunosuppressive corticoid-sparing agents may reduce these problems. Cortisone-induced types of diabetes are frequent in the aging population, and these patients must be screened before initiation of corticosteroid therapy. The intervention of a dietician must be proposed to set up an adapted diet in terms of carbohydrate intake and to prevent weight gain or low sodium retention with advice on caloric, sugar, and salt intake. To prevent cortisone myopathy, patients should be advised to practice regular physical activity (fast walking 30–45 min per day) and even have kinesiology therapy muscular strengthening sessions in the event of proven amyotrophy.

Examination by a psychiatrist may be useful for patients with a previous psychiatric history to evaluate the risk of psychiatric decompensation under corticoid and interferon-α treatment.

The importance of preventing cortisone-induced osteoporosis is often underestimated. This prevention aims to limit the risk of fractures in patients undergoing long-term corticosteroid therapy (> 7.5 mg/day of equivalent prednisone for > 3 months, in children > 0.3 mg/kg/day)). French recommendations were issued in 2014; they consider the cases of non-menopausal women and of men < 50 years old, the primary population affected by Behçet’s disease but for whom the need to prevent cortisone-induced osteoporosis is less evident than for older populations:

- Bone densitometry (DMO) is recommended for patients beginning long-lasting corticosteroid therapy (> 7.5 mg/day of equivalent prednisone during > 3 months) and in those undergoing corticosteroid therapy for > 3 months who did not have an initial evaluation by DMO.
- Screening for infra-clinical collapses by spinal x-rays must be carried out in cases of > 4 cm loss of height compared to the height at 20 years or > 2 cm loss of height during the follow-up.
- The use of the FRAX^®^ score (determination of the risk of fracture at 10 years) is not validated in non-menopausal women and men < 50 years old.
- The measure of bone remodeling markers is not relevant.
- The minimum effective dose of corticosteroid therapy is an important goal.
- Daily inputs of calcium, preferably via food, must be carefully monitored; systematic calcium supplementation is not, in fact, recommended.
- Measurement of 25-OH-vitamin D levels allows accurate compensation to maintain them above 30 ng/ml.
- Physical activity is essential.
- Smoking cessation is essential.
- Consumption of alcohol should be limited.

In cases of arterial lesions, blood pressure should be closely monitored in accordance with the recommendations in effect. Tobacco use is a risk factor for vascular complications, and cessation of tobacco use is required for all patients.

#### Mucocutaneous damage

Colchicine is the first-line treatment in the absence of contraindications to prevent the recidivation of mucocutaneous lesions, particularly buccal aphthae, genital lesions or those of erythema nodosum, at a posology usually between 1 and 2 mg/day. Bucco-dental hygiene is essential. Class I or II dermocorticoids may be proposed for the treatment of buccal and genital aphthosis even though short-term oral corticosteroid therapy is sometimes necessary. In cases of debilitating and resistant buccal aphthosis, a mouthwash with corticosteroids may be useful. Xylocaine in gel may be useful for painful genital ulcers.

In specific cases (refractory patients with severe mucocutaneous damage), other treatments, such as apremilast (inhibitor of phosphodiesterase 4), anti-TNFα, azathioprine, thalidomide, and ustekinumab, may be considered.

In a randomized phase II clinical test including 111 adult patients with recurrent aphthosis, apremilast (Otezla^®^) (30 mg × 2/day) significantly reduced the average number of oral ulcers per patient compared with placebo. Anti-acne treatments have limited effectiveness on pustular lesions.

#### Articular involvement

Colchicine is the first-line treatment at doses usually ranging between 1 and 2 mg/day. Intraarticular injections of corticosteroids are an additional interesting treatment in view of the oligoarticular damage which preferentially affects the large joints. NSAIDs or short-term treatments of oral prednisone may also be proposed as a treatment for an articular attack. In refractory and recurrent cases, DMARD treatment is necessary. Anti-TNFα, azathioprine (2 mg/kg/day), and methotrexate (0.3 mg/m^2^/week) may be proposed.

#### Ophthalmological involvement

Care and follow-up must be carried out with an ophthalmologist familiar with chronic inflammatory diseases of the eye. Ocular involvement leads to serious damage in BD and affects the functional prognosis. Relapses are frequent. A reduction (or cessation) of immunosuppressants or immunomodulating treatment may be considered only after a 2-year remission in the framework of severe Behçet’s disease, and an expert examination is advised.

In cases of anterior uveitis, corticosteroid therapy should be administered locally by eye drops containing dexamethasone, a powerful corticoid. The timing of administration should be proportional to the severity of the inflammation of the anterior segment. Clinical monitoring of local corticosteroid therapy must be close (at 48 h initially, then every 1–2 weeks, depending with the severity) while assessing the Tyndall effect of the anterior chamber and measuring the intraocular pressure systematically because of the frequency of cortisone-induced ocular hypertonia. Mydriatic and cycloplegic eye drops (tropicamide, neosynephrine, atropine) are often prescribed in association with topical corticosteroids to either inhibit posterior synechiae or prevent their formation. In case of ocular hypertonia, one or several hypotonic eye drops will be prescribed while avoiding, if possible, the analogues of prostaglandins because of their pro-inflammatory action. Uveitis with hypopyon generally requires systemic corticosteroid therapy. Some experts recommend the use of immunosuppressants (like azathioprine) for BD-related anterior uveitis if the subject is a young man and if the uveitis is a relapse.

Every posterior uveitis of BD must be treated with systemic corticoid treatment associated with immunosuppressants.

Systemic corticosteroid therapy is prescribed, according to the severity, at an oral dose of 0.5–1 mg/kg/day and may be preceded by intravenous pulses of methylprednisolone at the dose of 500 mg/day for 3 consecutive days. Azathioprine given at a dose of 2 mg/kg/day and cyclosporin at a dose of 3 mg/kg/day are good corticosteroid-sparing treatments and appear to be interesting for preventing relapses. They improve visual acuity by reducing posterior ocular damage of BD. The treatment does not appear to be sufficient for severe uveitis with reduction of visual acuity, macular edema, and/or retinal vasculitis, which constitute a therapeutic emergency.

In cases of flare of posterior uveitis with macular edema and/or retinal vascularity, intravenous infusions of infliximab at the dose of 5 mg/kg or subcutaneous injections of adalimumab (80 mg dose initially, then 40 mg/15 days) make it possible to obtain a rapid improvement in most cases. IFN-α (Roferon: 3 million units 3 times per week subcutaneously) may be proposed as an alternative therapeutic agent. Etanercept, on the other hand, is not indicated for the treatment of BD uveitis. Few data are currently available concerning certolizumab and golimumab for this indication.

IL-6 inhibitors may be effective in reducing post uveitis and macular edema in BD.

Recommendations for the care of ocular involvement in Behçet’s disease:

- The initial care and follow-up of patients with BD uveitis requires close collaboration with an ophthalmologist.
- Every patient with BD posterior uveitis must be treated by an immunosuppressant or biotherapy (azathioprine, cyclosporin A, interferon-α, or anti-TNFα).
- All severe sight-threatening involvement must be treated with high doses of corticosteroids associated with anti-TNFα or IFN-α.
- All systemic corticosteroid therapy must be associated with an immunosuppressant such as a corticoid-sparing agent.
- Intravitreous injections of corticosteroids associated with systemic treatment are practicable in the event of unilateral damage.
- Patients refractory to azathioprine or cyclosporin can be treated by either a monoclonal anti-TNFα or interferon α. The choice depends on the infectious risk of the patients (tuberculosis), their tolerance of IFN-α, and the experience of the clinician.
- Concerning isolated previous damage, the administration of systemic immunosuppressants may be considered in the event of factors with poor prognosis, such as young age, early onset of the disease, and male sex.

#### Neurological involvement

##### Parenchymatous forms

Treatment of parenchymatous forms is based on glucocorticoids, at high doses in the initial treatment (intravenous bolus of methylprednisolone, 500 mg/day for 3 days) followed by corticosteroid therapy at 1 mg/kg/day (without exceeding 80 mg/day) of equivalent prednisone for 3 weeks with a progressive decrease (15–20 mg/day at 3 months and ≤ 0.1 mg/kg/day at 6 months). Neurological involvement is a serious feature of BD that compromises the vital and functional prognosis, and it evolves with frequent relapses. Reduction (or cessation) of immunosuppressant and immunomodulating treatment can be considered only after at least 2 years of remission in the framework of severe Behçet’s disease. Expert examination is advised.

The treatment of isolated cases of meningitis is based on high-dose glucocorticoids. The immediate addition of an immunosuppressant (such as azathioprine) is not recommended during the first episode but may be considered in the event of a relapse while on corticosteroids.

Cyclosporin no longer has a role in treatment because of an increased risk of neurological aggravation.

##### Extraparenchymatous forms (cerebral thrombophlebitis)

The treatment of cerebral thrombophlebitis of BD is also based on the prescription of glucocorticoids at high doses for an attack and by intravenous pulses of methylprednisolone (500 mg/day during 3 days) followed by corticosteroid therapy at 1 mg/kg/day (without exceeding 80 mg/day) of equivalent prednisone for 3 weeks with a progressive decrease (15–20 mg/day by 3 months and ≤ 0.1 mg/kg/day by 6 months).

The immediate addition of an immunosuppressant (such as azathioprine) is not recommended during a first episode but may be considered in the event of a relapse while on corticosteroids.

The prescription of anticoagulants at a curative dose is recommended. The duration of the anticoagulation is disputed but is generally from 12 to 18 months. The risk of hemorrhage must always be evaluated, particularly to verify the absence of aneurysmal arterial lesions.

#### Vascular involvement

It is currently clearly established that immunosuppressant treatment is the cornerstone of the therapeutic strategy in these severe forms of BD. It is based on the fact that inflammation of the vascular wall probably plays a preponderant role in the occurrence of thrombotic vascular lesions.

Although no pro-thrombotic factor has been identified in BD and the pathogenesis of thrombosis is linked to endothelial inflammatory disease, the experts recommend the use of curative anticoagulation for deep vein thrombosis. Curative anticoagulation is recommended in venous thromboses after evaluation of the risk of hemorrhage and verification of possible aneurysmal arterial lesions. The duration of anticoagulant treatment will be from 3 to 6 months, except in serious forms, such as thromboses of the supra-hepatic veins and/or of the vena cava, which require extended anticoagulation.

Vascular involvement results in serious damage in BD; it is life-threatening, and relapses are frequent. Reduction (or cessation) of the immunosuppressant or immunomodulating treatment can be considered, except in exceptional cases, only after at least 2 years of remission in the framework of severe BD, and an expert opinion is advised.

Surgical or endovascular care (for aneurysm, severe aortic regurgitation, etc.) must not be delayed if the patient is symptomatic. It is mandatory to introduce corticosteroid and immunosuppressant treatment before surgical care to reduce the risk of postoperative complications. Postoperative complications are, in fact, significantly less frequent in patients receiving immunosuppressants and/or without systemic inflammation at the time of the vascular procedure. Multidisciplinary care at an expert center is indispensable for determining the optimal moment for the intervention. Long-term follow-up is necessary to detect complications and recurrences.

In cases of pulmonary aneurysms and the occurrence of hemoptysis, it is important to determine the role and mechanism of appropriately targeted interventional radiology in relation to either the pulmonary aneurysm (pulmonary vasocclusion) or perianeurysmal bronchial hypervascularization (bronchial arteriography with embolization). Surgery in the active phase of hemoptysis has a poor prognosis. Care should be carried out in multidisciplinary cooperation and at an expert center.

#### Digestive involvement

Digestive involvement in BD must be confirmed by endoscopy and/or imaging. Any digestive ulcerations connected with NSAIDs or related to infection must be eliminated.

In forms with digestive involvement and diarrhea and frequent secondary effects of colchicine on transit (diarrhea in 1–10%), use of colchicine must be discussed with a gastroenterologist. The treatment could, for example, be adapted after checking the intestinal inflammation.

#### Treatment in children

The drug-based therapeutic care of children is carried out based on the adult recommendations, given the small number of studies carried out in children. The choice of treatment is considered a function of the age, tolerance, and ease of use and is as economical as possible regarding long-term corticosteroid therapy, the effects of which are detrimental at all pediatric ages. The most complex patients, because of the severity of their BD or their difficult familial and social situations, require discussion in a multidisciplinary cooperation meeting with the reference/competence centers to ensure their best care.

#### Treatment algorithm

All the indicated posology is given. For severe damage, only induction treatment is indicated, while maintenance treatment must be adapted to the initial response and tolerance.

##### Objective

- Comfort of the patient and socio-professional maintenance
- Salvage therapy, functional or vital
- Prevention of outbreaks, serious complications, and sequelae
- Limitation of the treatment side-effects

##### Actors

- Evaluation: adult practice physicians, internal medicine specialists or rheumatologists or specialized pediatricians (reference/competence centers), ophthalmologists, dermatologists, neurologists, gastroenterologists, vascular surgeons, etc.
- Coordination: general physicians or pediatricians

##### Mucocutaneous involvement

- Symptomatic treatment of aphthae (Appendix 3)
- Topical corticosteroids or per os
- Colchicine 1–2 mg/day

Refractory:

- Apremilast (30 mg × 2/day)
- In the event of failure or intolerance: azathioprine (2 mg/kg/day) (Appendix 3, Box 1), anti-TNFα, thalidomide (50–100 mg/day), or ustekinumab.

##### Articular involvement

- Corticosteroid joint injection, NSAIDs, oral corticosteroids for short-term treatment
- Colchicine 1–2 mg/day

Refractory and/or recurrent:

- Azathioprine (2 mg/kg/day) (Appendix 3, Box 1), methotrexate (0.3 mg/kg/week) (Appendix 3, Box 2) or anti-TNFα.

##### Ocular involvement

- Isolated anterior uveitis:
- Eye drops: corticosteroids ± cycloplegic ± hypotonic
- Hypopyon: oral corticosteroids
- Poor prognosis (young man, recurrent form): azathioprine per os (Appendix 3, Box 1)
- Posterior uveitis:

General corticosteroid therapy: oral [according to severity, 0.5 to 1 mg/kg/day declining ± bolus (500 mg/day x 3 days) + immunosuppressants]

- Less severe forms: azathioprine (2 mg/kg/day) (Appendix 3, Box 1) or cyclosporin (3 mg/kg/day) per os
- Severe forms: anti-TNFα (infliximab IV, adalimumab SC) or INF-α (Roferon 3 million units × 3/week subcutaneous)

##### Neurological involvement

- Parenchymal: systemic corticosteroid therapy 1 mg/kg/day per os ± bolus IV (500 mg/day 3 days)

Serious form (Rankin score ≥ 2): cyclophosphamide IV (Appendix 3, Box 3) or anti-TNFα (infliximab). Moderate form (Rankin score < 2): azathioprine per os (2 mg/kg/day) (Appendix 3, Box 1) or methotrexate (0.3 mg/kg/week) (Appendix 3, Box 2)

If meningitis is isolated: systemic corticosteroid therapy solely as first-line treatment

- Extraparenchymal (cerebral thrombophlebitis):

systemic corticosteroid therapy 1 mg/kg/day per os ± bolus IV/3 day and effective anticoagulation ±

azathioprine per os (2 mg/kg/day) (Appendix 3, Box 1)

##### Vascular involvement

**Venous thrombosis, acute deep**

- **Severe forms (VC, Budd–Chiari):** Systemic corticosteroid therapy 1 mg/kg/day per os ± bolus (500 mg/day × 3 days), cyclophosphamide IV (15 mg/kg without exceeding 1.2 g) (Appendix 3, Box 2), or anti-TNFα (infliximab 5 mg/kg), effective anticoagulation the duration of which is to be discussed and associated with the anti-inflammatory treatment
- **Specific treatment of Budd–Chiari syndrome:** treatment of portal hypertension and interventional radiological treatment to be considered. Regarding the hepatological aspects, referral to an expert center.
- **Non-severe forms (of the limbs):** Systemic corticosteroid therapy 0.5 mg/kg/day per os and effective anticoagulation.
- **Recurrent forms:** azathioprine per os (2 mg/kg/day) (Appendix 3, Box 1) or anti-TNFα

**Arterial involvement**

Management and prophylaxis of cardiovascular risk factors (cessation of tobacco use, etc.).

- **Severe forms (pulmonary artery aneurysms, aorta, and/or multiple aneurysms, etc.):** systemic corticosteroid therapy 1 mg/kg/day per os ± bolus (500 mg/day × 3 days), cyclophosphamide IV (Appendix 3, Box 2), or anti-TNFα (infliximab).
- **Less severe forms (aneurysms peripheral):** systemic corticosteroid therapy 0.5–1 mg/kg/day and azathioprine per os (2 mg/kg/day) (Appendix 3, Box 1)

If symptomatic: surgery or interventional radiology always completed by medical immunosuppressant treatment. In case of symptomatic pulmonary aneurysm, always favor interventional radiology over surgery.

##### Digestive involvement

- Confirm the digestive involvement by endoscopy and/or imaging.
- Eliminate ulcerations induced by NSAIDs and of infectious origin.
- Systemic corticosteroid therapy 0.5 mg/kg/day, and azathioprine per os (2 mg/kg/day) (Appendix 3, Box 1) or 5-ASA.

In severe forms, indication for anti-TNFα treatment.

##### Pregnancy and lactation

Colchicine is not contraindicated and must be continued at the same doses during pregnancy and lactation. In serious forms of BD (vascular, neurological, etc.), pregnancy is not contraindicated if the disease has been in remission for at least 12 months. Azathioprine, cyclosporin, and anti-TNFα are authorized during pregnancy and lactation if their continuation is justified by BD status (Center for Teratogenic Agents: www.lecrat.fr).

##### Contraception

Estrogens are to be avoided in female patients presenting BD with vascular damage. Progestin and mechanical contraceptives should be favored.

### Therapeutic education and lifestyle modification (on a case-by-case basis)

A project for specific therapeutic education was proposed to the RHA in 2019 with the support of the French Association for Behçet’s disease. Certain transversal programs for therapeutic education may be addressed to patients affected by BD: transversal case developed in the FAI^2^R centers addressing the themes of transition and programs concerning certain treatments (particularly corticosteroid therapy and immunosuppressants). One of these programs is specifically dedicated to children and adolescents (TTP MIRAJE).

An annual review of TTP programs for rare diseases is available and updated regularly: www.etpdiseasesrare.com.

Caregivers for these patients, as well as their aides, must be informed about the importance of regularly taking the treatments prescribed, the risk of a suddenly stopping treatment, and the warning signs suggesting relapse or complications of BD.

### Transition from pediatric to adult care

The recommendations of the EULAR for the transition of patients with a rheumatological disease have recently been published. It is important that every department (pediatric and adult) has a written protocol on the transition, with a coordinating physician for the transition. Some transition consultations for adolescents must be set up to allow them to become autonomous in relation to their family and address specific points, such as fertility, pregnancy, genetic counseling, and therapeutic observance. A European group of experts has created a checklist of the topics to be addressed during the transition process to assist professionals.

The transition should be prepared several years in advance by the referring pediatrician and, as necessary, by a therapeutic education team with transition workshops. It is necessary to ensure that the patients thoroughly understands their disease and its treatments. It is important for patients to understand the program and to become autonomous: they will begin their personal encounters alone and will be examined without their parents in the consultation space.

The adult practice physician must be identified in advance. A consultation form for the transition must be sent by the pediatrician to the adult practice physician before the consultation.

Carrying out a common pediatrician/adult practice physician consultation is recommended. This consultation should last at least 45 min and be prepared in advance. If it was not possible to organize this consultation, then alternate consultations may be introduced.

The timing of the transition must be selected carefully. The disease must be inactive, and there must be no other risks for the patient during the same year.

The adult practice physician who was present during the transition consultation should not be changed. A pediatrician must also be available for the patients and their families. The timing of the consultations must be a little more frequent, and it is necessary to tell the patient that the pediatrician will be kept regularly informed. All of this aims to avoid an upsurge of outbreaks, departure from the course of treatment, and premature mortality.

All useful tools for this transition phase may be found and downloaded at the site of the FAI^2^R branch: www.fai2r.org/transition.

### Socio-professional and academic aspects and renewal of AID

The socio-professional or academic impact of the disease may be significant. Professional reclassification or invalidation may be necessary. In case of combination of the adjuvant treatment with corticosteroid therapy, a cessation of work is frequently required during the first 6 months of treatment.

In cases of development of a polyhandicap, it might be necessary to provide arrangements for daily life (housing, transportation, professional reclassification, etc.) and to provide specific help for filling out the medical portion of the application files for the MDPH.

For children in school, establishing a PIR (Project for Individualized Reception) with the director of the academic institution is strongly recommended.

Because of the duration of the conventional treatment and the extended risks of relapse, which require long-term monitoring, the assignment of long-term conditions can be carried out over a period of 5 years and should be renewable.

### Recourse to patient associations

Patient associations are useful for helping the patient and his/her relatives by offering listening and moral support so they do not become isolated. They make it possible to create connections among the patients, who can share their experiences. They may improve the patient’s course of health: information on pathology, long-term follow-up, access to the care network (competence centers, reference centers, rare diseases centers, etc.) and social services. They may improve cooperation among patients, caregivers, and the medical-social and administrative institutions.

## Follow-up

### Objective

- To ensure good control of disease activity and to detect and treat possible relapses and complications of BD;
- To ensure the observance and decrease of the treatment in patients in whom the disease activity is being monitored;
- To confirm tolerance of the treatment;
- To detect early and late complications of the disease or its treatment;
- To accompany the child, adolescent, and their families on a daily basis;
- To ensure the therapeutic training of the patient.

### Professionals involved (and modalities of coordination)

Follow-up is ideally coordinated by the physician having expertise in BD, most often an internal medicine specialist or a rheumatologist, ophthalmologist, pediatrician, or adult practice physician, and by the general physician or pediatrician who optimizes the coordination of the care proposed. Other specialized physicians may be brought into intervene in the follow-up of the patients: a dermatologist, neurologist, vascular physician, or gastroenterologist. The role of the general physician or pediatrician is essential in the first-line evaluation of the patient. Other health professionals may be called upon: professionals in therapeutic education, dietitians, state-certified nurses, physiotherapists, occupational therapists, psychologists, social welfare assistants, and so on.

### Timing and contents of the consultations

Disease activity is not easy to measure during the course of BD. This is particularly due to the developmental profile of BD, which has a chronic development with, in particular, an aphthosis and permanent or recurrent articular and cutaneous symptoms. This symptomatology may be interspersed with major events, such as uveitis, thrombosis, or authentic arthritis. There is no reliable biological marker to precisely evaluate the activity of BD. Inflammatory biological syndrome, as an elevation of the C-reactive protein (CRP) level, is not sensitive enough to be able to correlate with any low-noise activity. The frequency of follow-ups depends on the severity of BD. A regular follow-up every 3–6 months is generally recommended. An annual consultation when BD is quiescent is indispensable. In serious forms, patients are reviewed on a monthly basis at the beginning. In clinical practice, the appreciation of the activity of BD is essentially based on data from the examination and the clinical examination for the search for physical signs attesting to active disease. Assessment of the impact on the quality of life is essential to judge the need to carry out therapeutic adjustments; it may be based on simple questions or on generic questionnaires, taking into account the age and particularly the SF36, FACIT, or PEdQoL. A score measuring the specific quality of life for BD (Behçet’s disease quality of life, BD-QoL) also exists.

There are several activity scores for BD that may assist the clinician in proceeding to a complete evaluation of the disease during the course of follow-ups. These scores, such as the Behçet’s disease current activity index (BDCAI), are constructed based on a systematic evaluation of clinical symptoms that may be observed during the course of BD and which lead to a quantified activity result. For example, BDCAI evaluates the symptoms (such as aphthosis and cutaneous, articular, ocular signs, etc.) during the course of the last 4 weeks. The principal interest of these scores, however, is to make it possible to define groups of patients in the framework of clinical or therapeutic research studies. These cannot replace good knowledge of the disease, which is necessary for deciding whether a functional or objective manifestation can be considered a result of BD or not. The Behçet’s syndrome activity score (BSAS) is a measure of BD activity based entirely on evaluation by the patients themselves.

## Data Availability

Not applicable.

## Appendix 1: Pathologies presenting in the form of a Behçet-like syndrome with an elevated CRP

**Table:**

Name of the disease	Gene involved Protein	Transmission	Primary clinical signs
Deficiency of mevalonate kinase	*MVK* MVK	Autosomal recessive	Recurrent fevers (5–10 days), buccal aphthae, cervical adenopathies, abdominal pain, enteritis, oligoarthritis, cerebellar ataxia, bacterial infections
A20 Haploinsufficiency	*TNFAIP3* A20	Autosomal dominant	Recurrent fevers, buccal and genital aphthae, cutaneous ulcerations/abscesses, uveitis, particularly anterior, enterocolitis, autoimmunity and variable hypogammaglobulinemia
PAAND (pyrin-associated autoinflammation with neutrophilic dermatosis)	*MEFV* Pyrine	Autosomal dominant	Recurrent fevers, buccal aphthae, acne, pyoderma gangrenosum, suppurative hidrosadenitis
Syndrome PFIT periodic fever, immunodeficiency, and thrombocytopenia	*WDR1* WDR1	Autosomal recessive	Recurrent fevers, buccal and perianal inflammation, marked microstomy), cellulites, thrombopenia, opportunist infections
PFAPA syndrome periodic fever, aphthosis, pharyngitis, adenitis	No gene known	NA	Recurrent fevers (3-8 days), buccal aphthae, cervical adenopathies, abdominal pain
Crohn’s disease	*NOD2/CARD15* NOD2/CARD15	Autosomal recessive 10% of Crohn’s Disease	Extended fever, bipolar aphthosis, oligoarthritis or SPA, enterocolitis

## Appendix 2: Local treatments of painful aphthae

**Table:**

Properties	Name	Molecules	Dose	Max. dose	Precautions	Duration	Action
*Antalgic*							
Gel	Pansoral ointment or gel^®^	Choline chloride salicylate of cetalkonium	1–4 application/day	4/day	Children > 6 years, allergy to salicylates	5 days	Antalgic antiseptic—Attention: alcohol content
	Aftagel^®^	Zinc sulfate, lidocaine	1 application to be repeated, if necessary	2 applications/day		5 days	Antalgic, antiseptic
	Pyralvex^®^ gel	Root of oxalic salicylic acid	4 applications/day	4 applications/day	Children > 6 years, allergy to salicylates	5 days	Anti-inflammatory, antalgic
Gingival solution	Pyralvex^®^ solution	Root of oxalic salicylic acid	4 applications/day	4 applications/day	Children > 6 years, allergy to salicylates	5 days	Anti-inflammatory, antalgic
Cp	Aphthoral^®^	Chlorhexidine tetracaine and ascorbic acid	Child: 6-15 years 1 cp to be sucked, to be repeated at the end of 2 h Adult: 1 cp to be sucked, to be repeated at the end of 2 h	3 cp/day for the child and 4 cp/day for the adult	Child > 6 years. To be taken apart from meals or beverages. CI for pregnant or nursing women for aphthoral	5 days	Antiseptic with local anesthetic—trophic action on the conjunctive tissue
	Drill^®^	Chlorhexidine Tetracaine					Antiseptic with local anesthetic
	Lysopaine^®^	Lysozymes Cetylpiridinium	1 cp to be sucked, to be repeated at the end of 2 h	6 cp/day	> 6 years	5 days	Natural antiseptic and antibacterial
*Protector/healing agent*							
Gel	Bloxaphtha^®^ gel junior or adult	Hyaluronic acid	3 applications/day	3 applications/day	Children > 30 months	5 days	Anti-inflammatory, anti-edematous, healing agent
	Borostyrol^®^ gel	Hyaluronic acid	3 applications/day	3 applications/day	Children > 36 months	5 days	Anti-inflammatory, anti-edematous, healing agent
	Hyalugel^®^ adult/Ado	Hyaluronic acid	5 applications/day	5 applications/day	Children > 30 months for the gel Ado	5 days	Anti-inflammatory, anti-edematous, healing agent
Gingival solution	Urgo aphtha filmogen^®^	Cellulosic derivatives, carboxylic acid, mineral acid	1 application before meals	4 x/day	Child > 6 years and CI pregnant women	5 days	Antalgic, healing agent, protection
cp	Lyso-6^®^	Lysozymes pyridoxine	1 cp to be sucked, to be repeated at the end of 1 h	8 cp/day	> 6 years CI if treatment by levodopa	5 days	Natural antibacterial and healing agent
*Local treatments*							
1st line	ULCAR 1 g^®^ suspension drinkable in packets	Sucralfate	5 ml × 4/day (2 packets in 1 glass of water, while gargling 4 times per day (after meals), then spit out, or place directly on the lesions)	4 ×/day	> 14 years	5 days	Topical protective action
2nd line (if aphthosis severe) Corticosteroids or NSAIDs	Betneval 0.1 mg buccal^®^	Betamethasone	Slowly suck the tablets, without chewing or swallowing, up to complete disintegration	5–10/day	> 6 years. Mouthwashes. Do not swallow	Several days	Anti-inflammatory
	Dermoval^®^	Clobetasol propionate	Prompt application on the buccal mucous		> 6 years. Do not swallow	Several days	Anti-inflammatory
	Solupred^®^ effervescent	Prednisolone	Adult 60 mg child 15 mg in 250 ml of EPPI	3–4 ×/day	> 6 years. Mouthwashes. Do not swallow	Several days	Anti-inflammatory
	Aspegic	Acetylsalicylic acid	1 g	1 packet in 1/2 glass of water	> 6 years. Mouthwashes. Do not swallow	Several days	Anti-inflammatory

Recommendations in accordance with aphthae and ulcerations (Loic Vaillant 2016)

1. Antalgic + local treatment
2. Local antiseptic mouthwashes and/or protectors
3. Systemic treatments

**Table:**

Properties	Name	Molecules	Dose	Max. dose	Precautions	Duration
*Local antiseptics (avoid those with alcohol)*						
	Paroex^®^	Chlorexidine 0.12% + water purified	15 ml pure 2–3 ×/day		> 6 years. Keep in the mouth 1 min, then spit out. Do not drink or eat for 30 min in order for active ingredients to act	5 days
*Buccal hygiene*						
Mouthwashes with bicarbonate	Solucare^®^	Bicarbonate of sodium 1.4% solution	3 ×/day after every meal	3/day	> 6 years. Mouthwashes and/or cleaning of the tongue and buccal cavity with a foam rod	5 days
	Bicarome^®^	Bicarbonate of sodium 1.4% cp	1 cp in 60 ml of water 3 ×/day after every meal	3/day	> 6 years. Mouthwashes and/or cleaning of the tongue and buccal cavity with a foam rod.	5 days
	Bicarbonate Of Sodium Officinal^®^ Powder 250 g	Bicarbonate of sodium 1.4% powder	1 c of coffee in 1/2 glass of water 3 ×/day after every meal	3/day	> 6 years. Mouthwashes and/or cleaning of the tongue and buccal cavity with a foam rod.	5 days
Descaling and avoid: alcohol-based mouthwashes, lip stick with drying glycerin, lemon rods or solution containing lemon, crushed drugs, open gels placed directly in the mouth, aphthogenic foods: spices, sodas, vinegar, lemon, alcohol						

## Appendix 3: Primary treatments used during the course of Behçet’s disease

**Box 1. Modalities of administration of azathioprine**

**Table:**

Azathioprine is administered orally at the dose of 2 mg/kg/day in 1, 2, or 3 daily doses, without exceeding 200 mg/day (based on the therapeutic tests published) and with rounding to the higher multiple dose of 25 mg (e.g., for a 70 kg patient, the dose will be of 150 mg/day). This dose may be increased to 3 mg/kg/day by the physician if he or she judges this to be appropriate (in cases of partial response at 2 mg/kg/day), in the absence of a study having proven a better effectiveness of azathioprine at the dose of 3 mg/kg/day, however. The maximum dosage must not exceed 200 mg/day, whatever the weight of the patient. On the other hand, the physician may reduce the daily dose by 25 mg to 50 mg in case of a minor side effect to improve the digestive or hematological tolerance of the treatment. If this is not sufficient and/or if the side effect noted is serious, the treatment must be discontinued immediately. When making the decision to introduce azathioprine, the physician currently can use the recommendations of the Network National of Pharmacogenetics (RNGx) published in 2017. Caution concerning genetic deficiency in TPMT (thiopurine methyltransferase) and the risk of rapid development of a myelosuppression is present in the RCP of azathioprine. There has not, however, been any pharmacogenetic recommendation in the RCP, contrary to the case of the American RCP.	
The Clinical Pharmacogenetics Implementation Consortium and the RNGx recommend searching for a TPMT deficiency based on the identification of the allelic variants TPMT*2, TPMT*3A, TPMT*3B, and TPMT*3C or on the phenotyping of the TPMT, making possible the classification of individuals as a function of their metabolic capacity and proposing adaptation of doses as a function of TPMT status.	
No studies, however, have indicated that an adaptation of doses based on the genotypic study would allow reducing the risk of hematological events, particularly during the course of chronic inflammatory diseases of the intestine. Thus, performing this test does not preclude strict hematological monitoring, particularly during the first weeks of treatment.	
The concomitant prescription of a treatment for hypouricemia by allopurinol or febuxostat is contraindicated (it increases the medullary toxicity). If allopurinol or febuxostat cannot be discontinued, then another immunosuppressant must be chosen.	
Azathioprine is usually prescribed for a duration of 12–24 months (optimal length not defined).	
Biological monitoring will include the regular performance of a hemogram of the platelets and of the transaminases (ASAT or ALAT) every week during the first month, then every month for 3 months, and then every 3 months up to cessation.	

**Box 2. Modalities of administration of methotrexate**

**Table:**

In Behçet’s disease, methotrexate is usually prescribed at the dose of 0.3 mg/kg/week, orally or SC. If the clinical and biological tolerance is satisfactory, the dose could be increased to 20 and then 25 mg/week until reaching this dose at the end of 4–6 weeks, and this dose will be maintained up to the end of the treatment.	
Folic acid supplementation (preferably folinic acid, which is more expensive), at the dose of 10 mg/week, 48 h after taking methotrexate, is necessary to reduce the potential toxicity, particularly mucous and hepatic, and to improve the rate of therapeutic maintenance.	
The pre-therapeutic assessment must include: hemogram, platelet level, hepatic enzymes, creatinine clearance, and thoracic x-ray.	
The timing of monitoring after initiating treatment is not optional, but biological monitoring every week for 1 month, then every month for 3 months, and then every 3 months up to cessation is acceptable.	
Methotrexate is excreted by the kidneys, and its use is not recommended if the rate of glomerular filtration is < 30 ml/min. It must be reduced when accompanied by a dose reduction (from 7.5 to 20 mg/week) if the glomerular filtration rate is between 30 and 60 ml/min.	
The combination of methotrexate and sulfamethoxazole/trimethoprim increases the risk of hematological toxicity. This combination is not recommended. If it is prescribed, it must be with extreme care, and close monitoring must be carried out. In this situation, it is preferable to propose aerosols of 300 mg pentamidine every 21–28 days and even of atovaquone (750 mg × 2/day) for the prevention of pneumocystosis rather than sulfamethoxazole/trimethoprim.	
When the withdrawal phase of methotrexate is started, a 5 mg decrease of methotrexate every month is possible at the end of treatment before its cessation.	

**Box 3. Modalities of cyclophosphamide administration**

**Table:**

Precautions prior to the administration	
Preservation of fertility must be ensured, or at least proposed to the patients, for both women of reproductive age and men.	
Hydration prior to and during the infusion is indispensable. It is completed by the administration of Mesna (off-label and without certainty of its usefulness for doses of cyclophosphamide < 600 mg/m^2^ by bolus), administered during and after the infusion of cyclophosphamide: • 1/3 of the dose equivalent of cyclophosphamide (in mg) by IV channel at H0 • Then 2/3 of the dose by IV channel to the end of the infusion (90th min) • And 2/3 of the dose to H4, orally.	
When cyclophosphamide is delivered orally, Mesna may also be administered orally (daily equivalent dose in mg; orally, possibly off-label).	
The monitoring of treatment by cyclophosphamide is based on the NFS and the platelet count, creatininemia, and the search for hematuria at a minimum of: • Before every infusion • Every 2 weeks during the first 3 months • Then on a monthly basis in the event of continuation of treatment, orally	
If the polynuclear neutrophils are < 1.5 × 10^9^/l on the date scheduled for the bolus, then the dose will be reduced by 25% or even postponed (while attempting to not delay the treatment by more than 2 weeks, in which case another therapeutic must be considered).	
Schedule of administration of cyclophosphamide •	
During the course of the induction treatment by cyclophosphamide, a maintenance treatment must be started between 2 and 4 weeks after the last bolus of cyclophosphamide, whatever maintenance treatment that is selected. • The schedule recommended is the following: cyclophosphamide by bolus by IV channel prescribed at the dose of 0.7 g/m^2^ every 28 days, without exceeding 1.2 g by injection.	

## Appendix 4: Modified Rankin score

**Table:**

Value	Symptom
0	No symptoms
1	No incapacitation apart from symptoms: activities and autonomy preserved
2	Slight handicap: incapable of ensuring regular activities but autonomy preserved
3	Moderate handicap: need assistance but walking possible without assistance
4	Moderately severe handicap: walking and daily movements impossible without assistance
5	Major handicap: permanent bed rest, incontinence and permanent nursing care

## Abbreviations

NSAIDs: Non-steroidal anti-Inflammatory drugs
ALD: Long-term condition
AMM: Marketing authorization
RHA: Regional health agency
CeRemAIA: Reference Center for Autoinflammatory Diseases and Inflammatory Amylose
CPK: Creatine phosphokinase
CRP: C-reactive protein
DMARDS: Disease-modifying anti-rheumatic drugs
TTP: Therapeutic training of the patient
EULAR: EUropean League Against Rheumatism
FAI^2^R: Center for Rare Autoimmune and Autoinflammatory Diseases
FMF: Familial Mediterranean fever
G: Gauge
HLA: Human leucocyte antigens
HSP: Heat shock proteins
HTIC: Intracranial hypertension
IFN: Interferon
IL: Interleukin
MRI: Magnetic resonance imaging
CSF: Cerebrospinal fluid
BD: Behçet’s disease
BDP: Behçet’s disease—pediatric
MDPH: Departmental House for Handicapped Persons
MEFV: FMF gene
CIID: Chronic intestinal inflammatory disease
OCT: Optical coherence tomography
PIR: Project for individualized reception
PCR: Polymerase chain reaction
PFAPA: Periodic fever, aphthosis, pharyngitis, and adenitis
PL: Lumbar puncture
NDCP: National Diagnostic and Care Protocol
APLS: Anti-phospholipid syndrome
TDM: Tomodensitometry
TNF: Tumor necrosis factor
